# Supplementary material for: IFNγ-Producing B Cells Play a Regulating Role in Infection-Mediated Inhibition of Allergy
Source: Biology (Basel). 2023 Sep 20;12(9):1259. doi: 10.3390/biology12091259 (PMC10525206; doi:10.3390/biology12091259)
Supplement: Supplementary file 1 [file biology-12-01259-s001.zip › biology-2564196-supplementary.pdf]

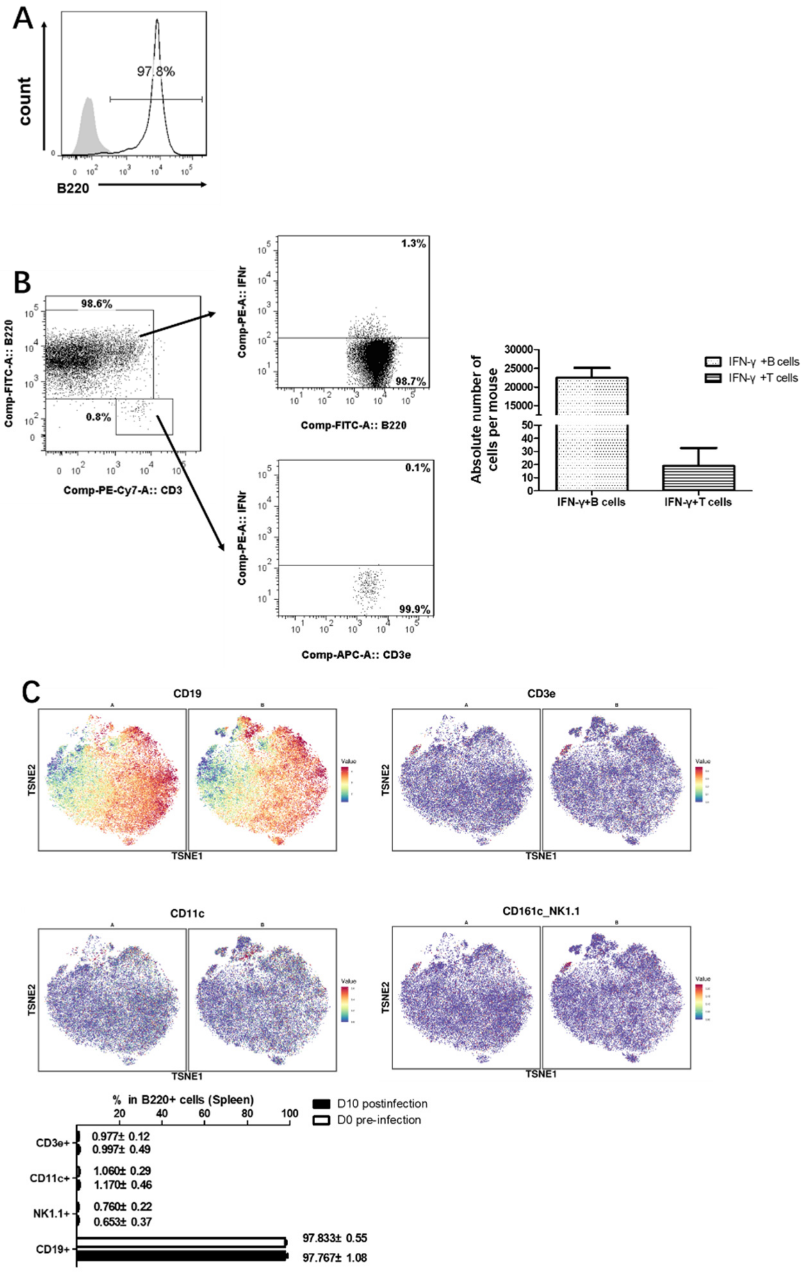

Supplementary Figure S1. The purity and the constitution of the isolated B220+ cells. B220+ cells were isolated and purified using MACS columns as described in Methods. (A) The purified cells were stained with anti-B220 mAb and detected by flow cytometry. (B) The percentage and absolute number of IFNγ+ cells were detected by intracellular staining of flow cytometry. (C) B220+ cells were analyzed for percentages of CD19+ cells, NK1.1+ cells, CD11c+ cells by flow cytometry. Data are shown as the mean ± SD of each group. Each group includes three or more mice. One representative experiment of three independent experiments is shown. *, $p<0.05$; **, $p<0.01$; ***, $p<0.001$.

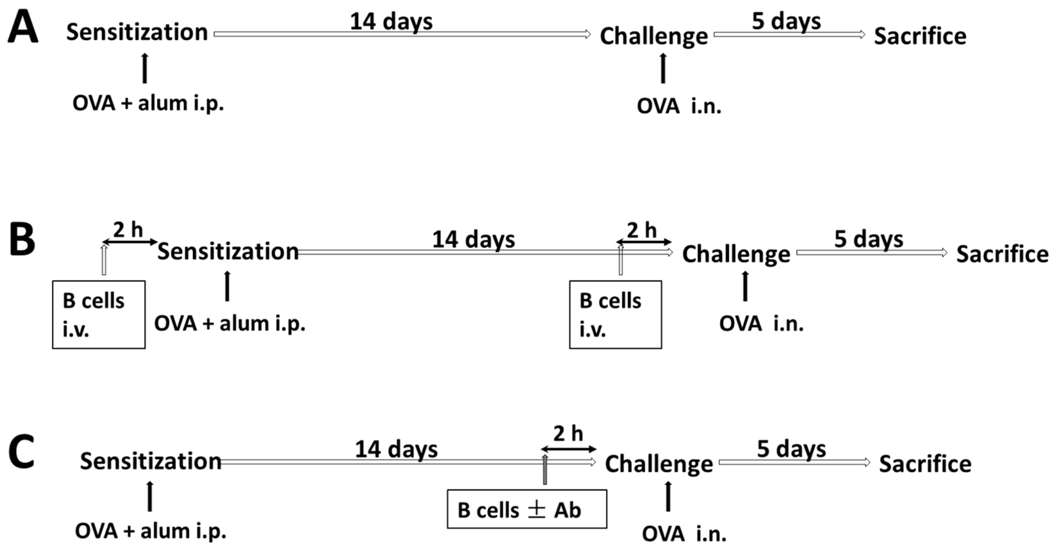

Supplementary Figure S2. Schedules of experimented design. (A) The basic model and the timeline of sensitization/challenge with OVA. (B) The timeline of B cell adoptive transfer. (C) The timeline of intranasal B cell transfer and simultaneous antibody treatment.

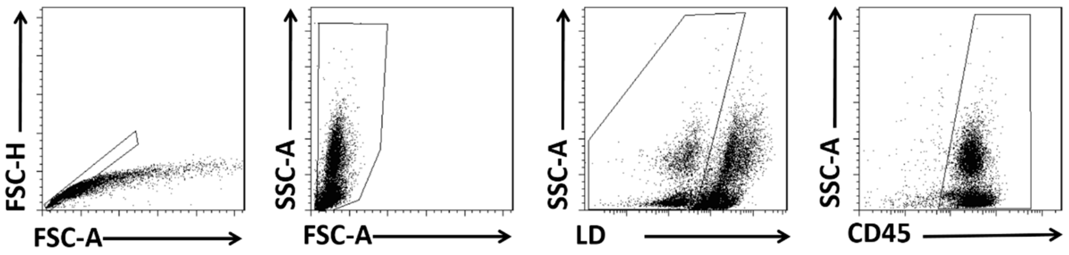

Supplementary Figure S3. The gating strategy for flow cytometry. Cells were prepared and stained with specific antibodies. FSC-H/FSC-A were used for gating single cell, SSC-A/FSC-A were then used for gating target cell population, followed by excluding LD^+ dead cells. $CD45^+$ cells were gated for leukocytes.
